# Development of an enzyme linked immunosorbent assay for detection of cyathane diterpenoids

**DOI:** 10.1186/s12896-014-0098-4

**Published:** 2014-11-18

**Authors:** Tian Shen, Lena M Hof, Heike Hausmann, Marc Stadler, Holger Zorn

**Affiliations:** Justus Liebig University Giessen, Institute of Food Chemistry and Food Biotechnology, Heinrich-Buff-Ring 58, Giessen, 35392 Germany; Justus Liebig University Giessen, Institute of Organic Chemistry, Heinrich-Buff-Ring 58, Giessen, 35392 Germany; Department Microbial Drugs, Helmholtz Centre for Infection Research, Inhoffenstrasse 7, Braunschweig, 38124 Germany

**Keywords:** Cyathane diterpenoids, ELISA, *Hericium erinaceus*, Striatal, Erinacine

## Abstract

**Background:**

So-called cyathane type diterpenoids are produced as secondary metabolites by basidiomycetes. Based on their antibacterial, fungicidal, and cytotoxic properties, cyathane type terpenoids represent interesting target compounds in fungal biotechnology.

**Results:**

An indirect competitive enzyme linked immunosorbent assay has been developed for detection of cyathane type diterpenoids. Rabbit polyclonal antibodies were raised against a mixture of striatal A and B conjugated to bovine serum albumin. The conditions for direct attachment of the hapten striatal B to a solid phase by passive adsorption were optimized. The cross reactivities of the striatals A, C and D, of the striatins A and B, and of the erinacines C and P to striatal B were determined. The validation study showed that the ELISA was precise and sensitive. The average IC_50_ of striatal B was 36.0 ng mL^−1^ with an inter-assay coefficient of variation (CV) of 13.2% (n = 5). Recoveries from striatal B spiked samples in the assay were in the range of 97.3 – 125.9%. A good correlation between the striatal B concentration measured by the ELISA and by HPLC-DAD (y = 1.1122× – 0.1585, R^2^ = 0.9942) was obtained from linear regression analysis. The suitability of the ELISA for detection of cyathane type diterpenoids in submerged cultures and fruiting bodies of *H. erinaceus* was studied. It showed cross reactivity with supernatants from submerged cultures and extracts thereof, but did not show cross reactivity with extracts from fruiting bodies.

**Conclusions:**

The developed method is appropriate for qualitative and quantitative detection of cyathane diterpenoids in complex mixtures. Due to its high sensitivity and specificity, it represents an ideal screening method for discovering new cyathane diterpenoids and new potential producers of them.

**Electronic supplementary material:**

The online version of this article (doi:10.1186/s12896-014-0098-4) contains supplementary material, which is available to authorized users.

## Background

**Figure 1:**
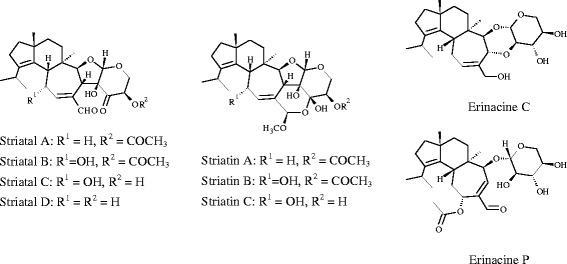
**Structures of striatals, striatins and erinacines.**

Cyathine and allocyathine were the first reported cyathane diterpenoids discovered by Allbutt and Ayer from a static liquid culture of the fungus *Cyathus helenae* (H. J. Brodie) in the early 1970s [1,2]. They were proven to be active against actinomycetes, Gram-positive and Gram-negative bacteria, and some fungi, including dermatophytes. Afterwards, various structurally related compounds, so-called cyathane type diterpenoids, were isolated from different basidiomycetous cultures, e.g. striatals and striatins from *Cyathus spp*., sarcodonins from *Sarcodon spp*., and erinacines from *Hericium spp*. (Figure 1) [3-7]. All of these compounds share a cyathane skeleton consisting of five-, six-, and seven membered rings and possess antibacterial, fungicidal, and cytotoxic properties. Fungal extracts prepared from *C. striatus* showed significant inhibitory effects on the NF-κB activation pathway and might be applied for cancer therapeutics [8]. Erinacines promote nerve growth factor (NGF) synthesis, which suggests the application of *H. erinaceus* or its secondary metabolites for the treatment and prevention of dementia and further neurodegenerative diseases [7,9-12].

Because of their interesting biological activities and their high potential in medicinal and pharmaceutical applications, cyathane diterpenoids have attracted increasing interest in recent years. At present, the most common method for analysis of cyathane diterpenoids is high performance liquid chromatography (HPLC) coupled to diode array and mass spectrometric detection [13].

As a rapid, sensitive and cost effective method, an ELISA specific for cyathane type secondary fungal metabolites may serve as a complementary method, especially in screenings for new producer strains. In the present study, an indirect competitive ELISA for the structure specific detection of cyathane type diterpenoids using polyclonal antibodies was developed, and its potential for the analysis of biological samples was proven.

## Results

### Optimum coating conditions

Striatal B was chosen as coating hapten in ELISA, because it is more polar than striatal A. In order to increase its solubility in aqueous solution, an equal volume of DMSO was added to the buffers. The optimum coating conditions (best signal-to-noise ratio and concentration dependence) were obtained by coating the plate in PBS/DMSO (1/1, v/v) buffer. More intense signals were obtained by coating at 24°C or 37°C, compared to coating at 4°C. Striatal B was adsorbed more efficiently by coating overnight than for 2 h. Based on these results, the optimum coating conditions for striatal B are summarized as follows: PBS/DMSO (1/1, v/v) buffer, and coating overnight at 24°C. The optimum concentration for coating of the hapten and the dilution of polyclonal antibodies (pAbs) were determined by checkerboard titration to be 5 μg mL^−1^ and 1:200, respectively, which were used in the following indirect competitive ELISA experiments.

### Tolerance against organic solvents

Due to the poor solubility of cyathane diterpenoids in aqueous solutions, the tolerance against organic solvents used to dissolve these compounds was tested for assay optimization. An addition of 5% DMSO, acetonitrile or methanol, respectively, to the pre-incubation solution was found acceptable. Methanol could be used up to 25% without a notable negative effect. High percentages of DMSO up to 30% and of acetonitrile up to 20% resulted in considerable negative effects on the assay. An addition of DMSO >30% or acetonitrile >20% showed a sudden increase of signals. Since methanol reacts with striatals [4], an addition of 5% DMSO or acetonitrile was selected for pre-incubation in the following indirect competitive ELISA.

### Cross reactivity

**Figure 2:**
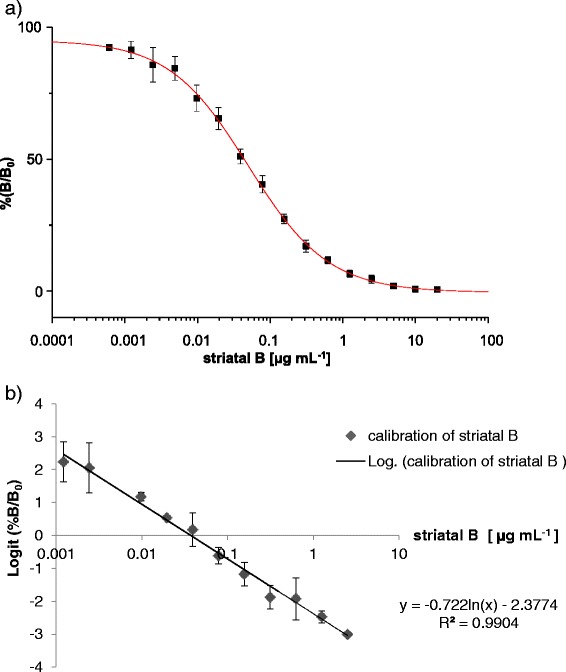
**Calibration graphs for the quantification of striatal B. (a)** Example of sigmoidal calibration curve for quantification of striatal B by indirect competitive ELISA. **(b)** Example of linearity of striatal B calibration curve. The linear working range was found to be 0.001 - 2.5 μg mL^−1^, y = −0.722 ln(x) – 2.3774, R^2^ = 0.9904.

Figure 2 illustrates a typical standard calibration curve of striatal B. The specificity of the pAbs against striatal B was evaluated by determination of the cross reactivities of several structurally related compounds, such as striatals A, C, and D, striatins A and B, and erinacines C and P. The IC_50_ values are given in Table 1. All of the tested cyathane diterpenoids showed cross reactivities against striatal B. No color reaction was detected with non-coated plates as blanks.

**Table 1:** **Cross reactivities of structurally related compounds to striatal B**

**Compounds**	Cross reactivity [%]*
Striatal B	100.0
Striatal A	39.8
Striatal C	77.8
Striatal D	74.4
Striatin A	24.1
Striatin B	109.3
Erinacine C	60.0
Erinacine P	21.1

*Related to molecular ratio.

### Assay precision

Intra-assay and inter-assay precisions were determined for several concentrations of striatal B (Table 2). The intra-assay precision was calculated from the extinction difference at 450 nm and 630 nm within one microtiter plate. Less than 10% CV were obtained in every experiment, indicating an acceptable precision. The inter-assay precision was obtained from %B/B_0_-values of 8 plates on different days. Variations of less than 20% were observed. The average IC_50_ of striatal B standard was 36.0 ng mL^−1^ with an inter-assay CV of 13.2% (n = 5).

**Table 2:** **Intra- and inter-assay precision**

**Concentration** **[μg mL** ^**−1**^ **]**	**Intra-assay (n = 3)** **CV [%]**	**Inter-assay (n = 8) CV [%]**
0.63	2.7	16.3
0.32	3.9	14.8
0.16	1.7	16.3
0.08	2.4	8.3
0.04	4.5	10.4
0.0006	5.1	3.6

### Recovery

**Figure 3:**
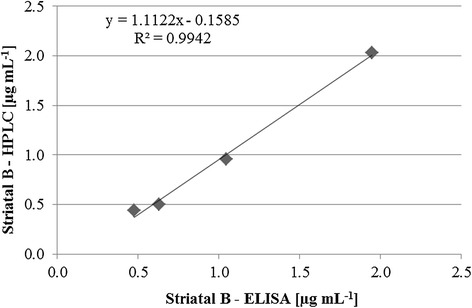
**Correlation between striatal B concentrations in spiked samples measured by ELISA (x-axis) and by HPLC-DAD (y-axis).**

To assess the assay accuracy, striatal B was added to the sample matrix in several concentrations, and the recoveries of the spiked samples were determined. In parallel, HPLC-DAD was used to analyze the spiked samples. The apparent recoveries of striatal B in the sample matrix as determined by indirect competitive ELISA ranged from 97.3% to 125.9%, while those obtained by HPLC-DAD varied from 95.8% - 110.2%. The limit of quantification was 0.02 μg mL^−1^ for ELISA, and 0.40 μg mL^−1^ for HPLC-DAD. A good correlation between the striatal B concentrations measured by ELISA and by HPLC-DAD (y = 1.1122× – 0.1585, R^2^ = 0.9942) was observed (Figure 3, Table 3).

**Table 3:** **Recovery of spiked striatal B in sample matrix**

**Spiked concentration [μg mL** ^**−1**^ **]**	**ELISA (n = 3)**		**HPLC-DAD (n = 2)**	
	**Detected [μg mL** ^**−1**^ **]**	**Recovery [%]**	**Detected [μg mL** ^**−1**^ **]**	**Recovery [%]**
10.010	out of calibration	-	10.50 ± 0.05	104.8%
5.005	out of calibration	-	5.26 ± 0.10	105.1%
2.002	1.949 ± 0.219	97.3%	2.03 ± 0.08	101.5%
1.001	1.048 ± 0.199	104.7%	0.96 ± 0.02	95.8%
0.501	0.630 ± 0.291	125.9%	0.50 ± 0.00	99.6%
0.400	0.478 ± 0.062	119.3%	0.44 ± 0.02	110.2%
0.200	0.252 ± 0.026	113.0%	n.d.	-
0.020	0.021 ± 0.001	102.8%	n.d.	-
0.001	n.d*	-	n.d.	-

*n.d: not detected.

### Analysis of *H. erinaceus*

**Figure 4:**
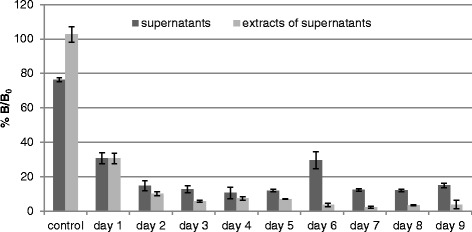
**Analysis of supernatants of submerged cultures of** ***H. erinaceus*** **and extracts thereof during 8 culture days.** Control: culture medium without *H. erinaceus.*

The indirect competitive ELISA was applied to samples prepared from submerged cultures as well as from fruiting bodies of *H. erinaceus*. The total concentrations of cyathane diterpenoids in supernatants and in extracts thereof are displayed in Figure 4. A similar change of %B/B_0_-values was observed in both cases during the 8 culture days. %B/B_0_-values in extracts of fruiting bodies from *H. erinaceus* were near to 100% and were independent from the sample concentration in the assay.

## Discussion

In the present study, striatal B was directly attached as a low molecular weight hapten to microtiter plates. In many studies the use of protein conjugated haptens for immobilization was reported [14-16], as the ability of proteins to adsorb to the plastic surface of microtiter plates is much higher than that of low molecular weight haptens. However, the coupling ratio of haptens to carrier proteins may vary depending on the coupling reaction and a waste of valuable haptens during conjugate synthesis is often unavoidable. The involvement of carrier proteins results in several problems. Firstly, it may cause unspecific binding to pAbs and thus reduce specific signals. Secondly, due to the large molecular dimension of carrier proteins, a shielding or masking effect may occur that impedes the binding of pAbs to the conjugated haptens [17]. Some researchers reported about pre-treatments of the plate surface, e.g. by UV irradiation or reagents in order to realize a direct binding of small molecules [18-20]. In the present study, the target analyte (molecular weight <500 g mol^−1^) was directly attached to microtiter plates without pre-treatment of the plate surface. Significant differences between the various coating procedures were found during the study of coating conditions. The pH of the coating buffer significantly affected the coating efficiency. The final pH of the coating buffer PBS, potassium acetate and sodium carbonate after mixing with DMSO was 9.7, 5.8 and 11.1, respectively. An influence of the pH on the coating efficiency has been reported in several publications [21-23]. In addition, a decomposition of striatal B was detected in potassium acetate buffer in a time course study by HPLC analysis (data not shown). That might be a reason for the poor attachment of striatal B to the plate in this buffer system. For striatal B, coating at pH 9.7 was more efficient than at pH 11.1.

The presence of organic solvents may influence the performance of an ELISA. Methanol caused the least negative effect, and the use of low concentrations of acetonitrile or DMSO was also acceptable. Similar results have been reported e.g. by [17,23,24]. The tolerance against organic solvents allows for the analysis of samples from fungal cultures which require extraction with an organic solvent. The sudden increase of the signals in the presence of acetonitrile and DMSO in high concentrations indicated a false positive signal possibly caused by denaturation of the antibodies and thus an unspecific adsorption to the blocking proteins.

All of the examined cyathane diterpenoids showed cross reactivities against striatal B. As expected, striatin B with IC_50_ = 109.3% showed the highest cross reactivity. Striatin B and striatal B differ only in the aldehyde group at the C_7_ ring, and this position of striatal B was used for BSA-conjugate synthesis for immunization. Therefore, striatin B and striatal B possess the same antigenic determinants. Erinacine P showed the lowest cross reactivity with IC_50_ = 21.1%. Based on the cross reactivities and molecular structures it might be suspected that the resulting antiserum displays the highest affinity toward the cyathane ring moiety.

The recovery experiments suggested that the developed ELISA is accurate and significantly more sensitive than the HPLC-DAD method. It could be used for the estimation of the total cyathane diterpenoid concentrations of biological samples. The assay showed interferences with constituents of the culture media of *H. erinaceus*, as a matrix effect was observed with the control samples (cf. Figure 4, control). Therefore, the use of organic extracts is preferred for the analysis of cyathane type diterpenoids in fungal culture supernatants. The product concentrations could be estimated during the cultivation period. However, the total concentrations represent the sum of all compounds showing cross reactivity against striatal B. They may thus differ from the individual concentrations determined by HPLC.

No significant interferences (%B/B_0_-values close to 100%) were observed with crude extracts of fruiting bodies of *H. erinaceus*. Therefore, the secondary metabolites present in the fruiting bodies, e.g. hericenone and erinacerine, did not cross react with the polyclonal antibodies. These results are in good agreement with a review article about secondary metabolites from *H. erinaceus* [12]. Up to date, erinacines have been mostly reported from submerged cultures of *H. erinaceus*, and only traces of erinacines have been found in fruiting bodies [25]. In the present study, the trace concentrations of erinacines in the fruiting bodies were apparently below the detection limit of the ELISA.

The CV% values for the inter-assay precision might be caused by many factors, e.g. the position of the wells in the plate (edge effect), the day to day variation in reagent preparation, the temperature of buffers, the variability in washing procedures, pipetting errors and the loss of enzyme activity of the secondary antibodies after prolonged storage [16,26].

## Conclusions

Polyclonal antibodies against striatal A/B were produced, and an indirect competitive ELISA was developed for determination of striatal B and structural related compounds. The method has shown satisfactory results concerning specificity, sensitivity and accuracy. The application to samples from submerged cultures and fruiting bodies of *H. erinaceus* indicated that the ELISA may be used for the estimation of total cyathane diterpenoid concentrations in complex mixtures. Due to its high sensitivity and specificity, it represents an ideal screening tool for discovering new potential producers of cyathane type diterpenoids. Further optimization will be needed to improve the inter-assay precision.

## Methods

### Chemicals and solutions

All reagents were of analytical grade unless specified otherwise. Ultra-pure water (produced by arium® 611 VF Water System, Sartorius, Göttingen, Germany) was used for all solutions, media, and HPLC eluents.

Acetonitrile (HPLC gradient grade), calcium carbonate, Edamin® K, 30% hydrogen peroxide (H_2_O_2_), and d-mannitol were purchased from Sigma Aldrich (Steinheim, Germany).

Salts, Agar-Agar, bovine serum albumin (BSA), EDTA, *α*-d(+)-glucose monohydrate, soy peptone, sucrose, 3,3’,5,5’-tetramethyl benzidine (TMB), Tween® 20, yeast extract, and organic solvents were purchased from Applichem (Darmstadt, Germany) or from Roth (Karlsruhe, Germany).

Malt extract was obtained from Fluka, Neu-Ulm, Germany. Molasses was obtained from Südzucker, Offstein, Germany. Oatmeal was provided from Dr. Oetker, Düsseldorf, Germany.

Standards of erinacines and striatals, originally isolated from the two selected producer strains, were obtained from the library of pure natural products of InterMed Discovery, Dortmund, Germany (IMD, formerly Bayer Healthcare) and their purity and identity was confirmed by 2D-NMR and HR-MS, prior to their use in the experiments. Additionally, striatals A and B (for immunization) and striatals C and D, striatins A and B, and erinacines C and P (for determination of cross reactivities) were extracted and purified from submerged cultures of the basidiomycetes *Cyathus striatus* and *Hericium erinaceus*. The structures were confirmed by comparison to the pure reference compounds and by NMR analysis (cf. Additional file 1: Supplementary material).

Solutions for ELISA:

Coating buffers:

1. 10 mM phosphate buffered saline (PBS), pH 7.4 mixed with DMSO, 1/1, v/v.
2. 0.1 M potassium acetate buffer, pH 4.0 mixed with DMSO, 1/1, v/v.
3. 0.1 M sodium carbonate buffer, pH 9.6 mixed with DMSO, 1/1, v/v.

Washing buffer: 10 mM PBS, pH 7.4 with 0.05% Tween® 20 (PBST).

Blocking reagent (1% gelatin) was purchased from Roche Diagnostics (Mannheim, Germany). Secondary antibodies (goat anti-rabbit lgG peroxidase conjugate) were obtained from Merck (Darmstadt, Germany).

Color development: 0.12 mg mL^−1^ TMB and 0.05% H_2_O_2_ in 0.05 M sodium acetate solution, pH 4.5 (TMB solution). This solution was freshly prepared before use.

Stopping reagent: 0.5 M sulfuric acid.

### Fungal strains

*Cyathus striatus* (STMA07048, isolated from basidiospores of a specimen from a trunk of Picea collected in July 1997 in Stelzenberg, Rheinland-Pfalz, Germany) and *Hericium erinaceus* (FU70034, isolated from basidiocarp tissue) for submerged culture were obtained from IMD. Prior to the experiments, the identity of both strains was confirmed by microscopic studies and by comparison of their ITS nrDNA sequences with reference data in Genbank. The fungi were maintained on a solid medium containing 20 g L^−1^ malt extract and 15 g L^−1^ Agar-Agar.They are deposited in the culture collection of IMD Natural Solutions GbR (formerly InterMed Discovery GmbH under liquid nitrogen. A duplicate strain and the corresponding specimen of the *C. striatus* material is also maintained at the personal herbarium and culture collection of Marc Stadler.

Fruiting bodies of *Hericium erinaceus* were obtained from a commercial provider www.pilzgarten.de, Helvesiek, Germany.

### Culture media

Soy peptone, yeast malt, and sugar molasses media were prepared according to [25].

### Instruments

#### HPLC-DAD

The HPLC system was from Merck Hitachi, Darmstadt, Germany, and comprised a pump (L-7100), an auto sampler (L-7200), an interface (D-7000), and a diode array detector (L-7455, 200 – 600 nm). Column: reversed phase, Nucleosil® 100–5 C_18_, CC 125/3 mm with a guard column Nucleosil® 100–5 C_18_, CC 8/3 mm (Macherey Nagel, Düren, Germany).

Flow rate: 0.4 mL min^−1^ for striatal and striatin analysis and 0.6 mL min^−1^ for erinacine analysis.

Eluent: Acetonitrile (A) and ultra-pure water (B).

Wavelength: 210, 233 and 254 nm.

Gradient for striatal and striatin: 50% A (0 min) – 94% A (14 min) – 94% A (17 min) – 100% A (19 min) – 100% A (25 min) – 50% A (30 min) – 50% A (40 min).

Gradient for erinacine: 30% A (0 min) – 50% A (15 min) – 50% A (16 min) – 100% A (23 min) – 100% A (38 min) – 30% A (43 min) – 30% A (47 min).

### Preparative HPLC

A semi-preparative HPLC system (Young Lin, Hongye Anyang, Korea) equipped with quaternary pump (YL9110S) and dual wavelength UV/Vis detector (YL 9120S), combined with a fraction collector (CHF 122SC, Advantec, Osaka, Japan) was used. A column Kromasil 100 C_18_, 7 μm, 250 × 20 mm (MZ Analysentechnik, Mainz) with a guard column Kromasil 100 C_18_, 7 μm, 50 × 20 mm (MZ) was used to isolate the target compounds.

Flow rate: 15 mL min^−1^.

Eluent: Acetonitrile (A) and ultra-pure water (B).

Gradient for striatals and striatins: 50% A (0 min) – 50% A (10 min) – 80% A (55 min) – 80% A (70 min) – 100% A (80 min) – 100% A (95 min). Wavelengths: 210 and 254 nm.

Gradient for erinacines: 25 A% (0 min) – 25% A (10 min) – 48% A (35 min) – 48% A (45 min) – 55% A (52 min) – 55% A (60 min) – 100% A (90 min) – 100% A (110 min). Wavelengths: 210 and 233 nm.

### ELISA

The ELISA was performed in microtiter plates Immuno Plate Maxisorp F96 (Nunc, Denmark). Incubation steps and color development were performed in an incubator INE 500 (Memmert, Schwabach, Germany). Washing was done by an eight-channel pipette Research Pro 50–1200 μL (Eppendorf, Wesseling-Berzdorf, Germany). The extinction was measured by a microtiter plate reader Synergy 2 (BioTek, Bad Friedrichshall, Germany).

### Biosynthesis and isolation of cyathane diterpenoids

#### Submerged cultivation

Pre-cultures of *C. striatus* were grown submerged in 250 mL Erlenmeyer flasks containing 100 mL soy peptone medium at 24°C and 150 rpm for 7 days. After that, the mycelium was homogenized with an Ultra-Turrax (IKA, Staufen, Germany) at 10,000 rpm for 10 sec. 40 mL homogenized mycelium were inoculated into a new 1,000 mL Erlenmeyer flask containing 400 mL soy peptone medium and incubated at 24°C and 150 rpm for further 12 days.

Pre-cultures of *H. erinaceus* were grown submerged in 250 mL Erlenmeyer flasks containing 100 mL yeast malt medium at 24°C and 150 rpm for 7 days. Afterwards, 40 mL homogenized mycelium were inoculated into 400 mL sugar molasses medium in 1,000 mL Erlenmeyer flasks and incubated at 24°C and 150 rpm for further 4 days to obtain erinacine P or 12 days to obtain erinacine C.

### Extraction and isolation

After submerged cultivation, the mycelia from 2.2 L culture media were separated from the supernatants by centrifugation at 2,880 *g* and 4°C for 10 min (Allegra® X-15R, BECKMAN COULTER™, Krefeld, Germany), and extracted twice with 400 mL ethyl acetate. The combined organic extracts were dried over sodium sulfate, and afterwards evaporated to dryness. A brown residue was obtained after evaporation, which was dissolved in acetonitrile and subjected to preparative HPLC. The extracts of *Cyathus striatus* contained the striatals A, B, C and D. The striatins A and B were prepared by conversion of the striatals A and B by stirring in methanol at room temperature overnight, respectively. The erinacines P and C were isolated from the extracts of *Hericium erinaceus*.

### Production of polyclonal antibodies

The aldehyde function of the striatals and NH_2_ functions of BSA were used for synthesis of striatal A/B (1/1, w/w)-BSA conjugates. The ratio of striatals to BSA was determined by MALDI-TOF-MS. About 17 striatal molecules were coupled to one BSA molecule (Eurogentec S.A, Seraing, Belgien). The production of rabbit pAbs against striatal A/B was performed by Eurogentec S.A. Two rabbits with ID SA6928 and SA6929 were immunized four times with 200 μg of the striatal A/B-BSA conjugate on day 0, 14, 28 and 56. An additional immunization was done on day 91with 400 μg striatal A/B-BSA conjugate. The final antisera were collected on day 115 and stored at −20°C without further purification until use. The pre-bleed sera of these two rabbits on day 0 before immunization were used as negative control in ELISA. The final antiserum from rabbit SA6928 was used in the following assays because of its higher specificity against striatal B compared to SA6929 (data not shown).

### Development of an indirect competitive ELISA

#### Optimization of coating conditions

Striatal B was directly attached to the plate in several different concentrations. The optimal conditions (buffer, temperature and time) for direct coating of striatal B were studied by an indirect non-competitive ELISA. The incubation temperatures were 4°C, 24°C and 37°C, and the coating time was 2 h or overnight.

### Checkerboard titration

The optimal dilution of pAbs and the optimal coating amount of striatal B were determined simultaneously by checkerboard titration (a two-dimensional titration method) by indirect non-competitive ELISA. Striatal B with different concentrations (0.04 to 20 μg mL^−1^, two fold dilution) was coated to the microtiter plate and then bound to pAbs with serial dilutions (1:50 to 1:6,400, stepwise in two fold dilution).

### Tolerance against organic solvents

The tolerance against organic solvents (DMSO, acetonitrile, methanol) used to dissolve the cyathane diterpenoids was examined by indirect competitive ELISA. Different proportions of organic solvent (5 - 50%, v/v, 0% as blank) were added to PBST with a final dilution of pAbs 1:200 and pre-incubated without competing molecules at 24°C for 1 h. The effects of organic solvents on the ELISA system were evaluated by comparing the measured extinctions to those of the blanks.

### ELISA

#### Coating

100 μL of 5 μg mL^−1^ striatal B in coating buffer 1 were pipetted in wells of a microtiter plate. 100 μL of 1 μg mL^−1^ BSA diluted in PBS were pipetted in two wells as positive control. The incubation was performed overnight at 24°C.

#### Blocking

200 μL of 1% gelatin were added to the wells and incubated at 24°C for 3 h.

#### Pre-incubation

Serial dilutions of analyte were prepared in a concentration range of 0.0003 - 20 μg mL^−1^ in PBST containing 5% acetonitrile or DMSO and a final pAbs dilution of 1:200. The incubation was performed for 1 h at 24°C in 2 mL Eppendorf® cups.

#### Incubation with pAbs

100 μL pre-incubated analyte were added to each well (in triplicate) and incubated for 1 h at 24°C. For the indirect non-competitive ELISA, 100 μL pAbs with a 1:100 dilution in PBST were used without competitors instead of the pre-incubated analyte.

#### Incubation with secondary antibodies

100 μL secondary antibodies with a 1:5,000 dilution in PBST were added to each well and incubated for 1 h at 24°C.

#### Color development

100 μL of the TMB solution were added to each well and incubated for 15 min at 24°C. Without washing step, 100 μL of 0.5 M sulfuric acid were added to each well to stop the color development.

#### Measurement

The extinctions were measured immediately by a microtiter plate reader at 450 nm and 630 nm.

Unless otherwise specified, the wells were washed with 300 μL washing buffer PBST three times after each incubation step. The indirect non-competitive ELISA involved all of the steps of the indirect competitive ELISA, except for the pre-incubation of pAbs with competitors.

### Data analysis

Striatal B standards and samples were analyzed in triplicates. The differences between extinctions at 450 nm and 630 nm were used for calculation and plotting the sigmoidal curves. %B/B_0_-values for calibration curves were calculated as follows:

$$ \%\frac{B}{B_0}=\frac{\varDelta E-\varDelta {E}_{excess}}{\varDelta {E}_0-\varDelta {E}_{excess}}\times 100 $$

*∆E*: the extinction differences of samples at 450 nm and 630 nm.

*∆E*_*0*_: the extinction differences of the upper asymptote of the sigmoidal curve between 450 nm and 630 nm.

*∆E*_*excess*_: the extinction differences of the lower asymptote of the sigmoidal curve between 450 nm and 630 nm.

### Cross reactivity (IC_50_)

The assay specificity was evaluated by obtaining sigmoidal curves for several structurally related compounds (striatals A, C and D, striatins A and B, and erinacines C and P) as competitors against the striatal B standard. The calibration curve of striatal B standard was measured individually for each plate. The IC_50_ were estimated by using a linearization of the calibration curves with a logit-transformation of %B/B_0_-values:

$$ logit\ \left(\%\ B/{B}_0\right)= ln\ \left(\frac{\%\ B/{B}_0}{100-\%\ B/{B}_0}\right) $$

Cross reactivity (IC_50_) = A/B × 100%.

A: concentration of striatal B standard at logit (%B/B_0_) =0.

B: concentration of competitors at logit (%B/B_0_) =0.

The linear regression of logit (%B/B_0_) was used for determination of concentrations in unknown samples.

### Recovery

Acetonitrile was spiked with striatal B at several concentrations (0.001 - 10 μg mL^−1^) and analyzed by indirect competitive ELISA as well as by HPLC-DAD. The concentrations of striatal B of spiked samples were calculated from linear regression of logit (%B/B_0_) in ELISA and linear calibration for striatal B in HPLC-DAD. The recoveries of striatal B were obtained by:

$$ recovery\ \left[\%\right]=\frac{\mathrm{detected}\ \mathrm{concentration}}{\mathrm{spiked}\ \mathrm{concentration}} \times 100\% $$

### Analysis of *H. erinaceus* samples

The supernatants and extracts of supernatants from submerged cultures, as well as extracts of fruiting bodies of *H. erinaceus* were analyzed by indirect competitive ELISA:

### Sample preparation for pre-incubation of supernatants in ELISA

50 μL of supernatants from submerged cultures (culture day 1 to day 8) were added to 950 μL PBST with a final DMSO proportion of 5% and a final pAbs dilution of 1:200. Culture medium without *H. erinaceus* was used as negative control.

### Sample preparation for pre-incubation of extracts of supernatants

20 mL of supernatants from submerged cultures (culture day 1 to 8) were extracted once with 20 mL ethyl acetate. 15 mL of the ethyl acetate phase was evaporated to dryness, and the residues were redissolved in 1 mL acetonitrile for ELISA and HPLC-DAD analysis. 5 μL of the extracts were added to 995 μL PBST with a final acetonitrile proportion of 5% and a final pAbs dilution of 1:200. Extracts of culture media without *H. erinaceus* were used as negative controls.

### Sample preparation for pre-incubation of extracts of fruiting bodies

32.6 g fruiting bodies of *H. erinaceus* were homogenized and then extracted with 200 mL ethyl acetate under stirring overnight. The ethyl acetate phase was dried over sodium sulfate and evaporated to dryness after filtration. 1 mg crude extract was dissolved in 1 mL acetonitrile. Several concentration levels (50.0, 10.0, 5.0, 2.5, 1.0 and 0.5 μg mL^−1^) of the extracts were used in the pre-incubation solution. Acetonitrile was used as blank.

### Availability of supporting data

The data sets supporting the results of this article are included within the article and its additional file.

## Additional file

Additional file 1: **Supplementary material.**
