# Supplementary material for: Development of an enzyme linked immunosorbent assay for detection of cyathane diterpenoids
Source: BMC Biotechnol. 2014 Nov 18;14:98. doi: 10.1186/s12896-014-0098-4 (PMC4239385; doi:10.1186/s12896-014-0098-4)
Supplement: Supplementary file 1 — Supplementary material. [file 12896_2014_98_MOESM1_ESM.docx]

### NMR

NMR spectra were recorded on a Bruker Avance III 600 MHz NMR spectrometer equipped with a 5 mm BBO Z-gradient probe. The data were collected and processed by TOPSPIN software (Bruker) running on a PC with Microsoft Windows^XP^. The NMR experiments were performed using Bruker standard pulse sequences and parameters. Chemical shifts are reported in ppm (δ scale) using the solvent signal as standard and coupling constants (J) in Hz.

Methanol-d_4_, ≥ 99,8% and chloroform-d_1_, 99,8 atom % D were purchased from Deutero, Kastellaun, Germany and Acros Organics, Geel, Belgium, respectively.

**Striatal A:**

^1^H NMR (CDCl_3_): δ (ppm): 0.97 (CH_3_, d, J = 7 Hz, 19-H or 20-H), 0.98 (CH_3_, d, J = 7 Hz, 19-H or 20-H), 0.99 (CH_3_, s, 16-H), 1.01 (CH_3_, s, 17-H), 1.53 (CH_2_, m, 8-H), 1.55 – 1.63 (CH_2_, m, 1_a,b_-H), 1.60 (CH_2_, m, 7-H), 2.16 (CH_3_CO_2_, s), 2.27 (CH_2_, m, 2-H), 2.38 (CH, d, J = 11.6 Hz, 5-H), 2.71 – 2.84 (CH_2_, m, 10_a,b_-H), 2.74 (CH, m, 18-H), 3.40 (CH, dm, J = 11 Hz, 13-H), 3.98 – 4.34 (CH_2_, dd, J = 12 Hz, 4.5 Hz; dd, J = 12 Hz, 4.5 Hz, 5’_a,b_-H), 4.14 (CH, d, J = 11 Hz, 14-H), 4.85 (CH, t, J = 4.5 Hz, 4’-H), 5.33 (CH, s, 1’-H), 6.97 (CH, dt, J = 8.6 Hz, 2.4 Hz, 11-H), 9.25 (CHO, s, 15-H)

^13^C NMR (CDCl_3_): δ (ppm): 17.5 (CH_3_, C-16), 20.5 (*C*H_3_CO_2_), 21.5 (CH_3_, C-19 or C-20), 21.9 (CH_3_, C-19 or C-20), 24.6 (CH_3_, C-17), 26.9 (CH_2_, C-7), 27.2 (CH, C-18), 28.5 (CH_2_, C-2), 29.3 (CH_2_, C-10), 36.4 (CH_2_, C-8), 38.3 (CH_2_, C-1), 41.9 (C-q, C-6), 42.8 (CH, C-5), 46.5 (CH_,_ C-13), 49.6 (C-q, C-9), 65.2 (CH_2_, C-5’), 75.2 (CH, C-4’), 84.0 (C-q, C-2’), 87.4 (CH, C-14), 108.3 (CH, C-1’), 136.1 (CH, C-4), 139.9 (C-q, C-3), 142.4 (C-q, C-12), 158.0 (CH, C-11), 170.5 (CH_3_*C*O_2_), 196.0 (CHO, C-15), 201.0 (C-q, C-3’)

**Striatal B:**

^1^H NMR (CDCl_3_): δ (ppm): 0.96 (CH_3_, s, 17-H), 0.98 (CH_3_, d, J = 6.7 Hz, 19-H or 20-H), 1.05 (CH_3_, d, J =6.7 Hz, 19-H or 20-H), 1.24 (CH_3_, s, 16-H), 1.51 – 1.69 (CH_2_, m, 1_a,b_-H), 1.57 (CH_2_, m, 8-H), 1.59 (CH_2_, m, 7-H), 2.15 (CH, m, 5-H), 2.17 (CH_3_CO_2_, s), 2.30 (CH_2_, m, 2-H), 3.01 (CH, sept, J = 6.7 Hz, 18-H), 3.33 (CH, dd, J = 10.5 Hz, 2.8 Hz, 13-H), 4.02 – 4.41 (CH_2_, dd, J = 12 Hz, 4.5 Hz; dd, J = 12 Hz, 4.5 Hz, 5_a,b_’-H), 4.66 (CH, d, 10.5 Hz, 14-H), 4.89 (CH, t, 4.5 Hz, 4’-H), 4.93 (CH, d, 7.5 Hz, 10-H), 5.38 (CH, s, 1‘-H), 6.89 (CH, dd, 7.5 Hz, 2.8 Hz, 11-H), 9.31 (CHO, s, 15-H)

^13^C NMR (CDCl_3_): δ (ppm): 20.6 (*C*H_3_CO_2_), 21.2 (CH_3_, C-16), 21.6 (CH_3_, C-19 or C-20), 21.9 (CH_3_, C-19 or C-20), 23.9 (CH_3_, C-17), 26.4 (CH, C-18), 28.8 (CH_2_, C-2), 28.9 (CH_2_, C-7), 36.7 (CH_2_, C-8), 39.3 (CH_2_, C-1), 42.5 (C-q, C-6), 45.9 (CH, C-5), 47.1 (CH_,_ C-13), 49.2 (C-q, C-9), 65.3 (CH_2_, C-5’), 68.8 (CH, C-10), 75.3 (CH, C-4’), 84.0 (C-q, C-2’), 86.8 (CH, C-14), 108.3 (CH, C-1’), 135.1 (CH, C-4), 140.1 (C-q, C-3), 143.6 (C-q, C-12), 153.6 (CH, C-11), 170.7 (CH_3_*C*O_2_), 196.5 (CHO, C-15), 201.1 (C-q, C-3’)

**Striatal C:**

^1^H NMR (CDCl_3_): δ (ppm): 0.95 (CH_3_, s, 17-H), 0.97 (CH_3_, d, J = 6.7 Hz, 19-H or 20-H), 1.04 (CH_3_, d, J = 6.7 Hz, 19-H or 20-H), 1.22 (CH_3_, s, 16-H), 1.51– 1.70 (CH_2_, m, 1_a,b_-H), 1.57 (CH_2_, m, 8-H), 1.59 (CH_2_, m, 7-H), 2.14 (CH, m, 5-H), 2.31 (CH_2_, m, 2-H), 3.02 (CH, sept, J = 6.7 Hz, 18-H), 3.26 (CH, dd, J = 10 Hz, 2.4 Hz, 13-H), 3.77 – 4.28 (CH_2_, dd, J = 12 Hz, 4.7 Hz; dd, J = 12 Hz, 3.7 Hz, 5_a,b_’-H), 4.03 (CH, m, 4’-H), 4.52 (CH, d, J = 10 Hz, 14-H), 4.93 (CH, d, J = 7.4 Hz, 10-H), 5.27 (CH, s, 1’-H), 6.90 (CH, dd, J = 7.4 Hz, 2.4 Hz, 11-H), 9.32 (CHO, s, 15-H)

^13^C NMR (CDCl_3_): δ (ppm): 21.1 (CH_3_, C-16), 21.6 (CH_3_, C-19 or C-20), 21.9 (CH_3_, C-19 or C-20), 23.9 (CH_3_, C-17), 26.4 (CH, C-18), 28.2 (CH_2_, C-2), 28.9 (CH_2_, C-7), 36.6 (CH_2_, C-8), 39.2 (CH_2_, C-1), 42.2 (C-q, C-6), 45.9 (CH, C-5), 46.0 (CH_,_ C-13), 49.2 (C-q, C-9), 68.6 (CH_2_, C-5’), 68.7 (CH, C-10), 75.0 (CH, C-4’), 83.6 (C-q, C-2’), 86.6 (CH, C-14), 108.1 (CH, C-1’), 135.0 (CH, C-4), 140.2 (C-q, C-3), 143.1 (C-q, C-12), 154.7 (CH, C-11), 197.0 (CHO, C-1), 204.3 (C-q, C-3’)

**Striatal D:**

^1^H NMR (CDCl_3_): δ (ppm): 0.97 (CH_3_, d, J = 6.8 Hz, 19-H or 20-H), 0.99 (CH_3_, d, J = 6.8 Hz, 19-H or 20-H), 0.99 (CH_3_, s, 16-H), 1.01 (CH_3_, s, 17-H), 1.53 (CH_2_, m, 8-H), 1.55 – 1.623 (CH_2_, m, 1_a,b_-H), 1.60 (CH_2_, m, 7-H), 2.27 (CH_2_, m, 2-H), 2.39 (CH, d, J = 11.5 Hz, 5-H), 2.75- 2.84 (CH_2_, m, 10_a,b_-H), 2.73 (CH, m 18-H), 3.34 (CH, dm, J = 10.6 Hz, 13-H), 3.68 – 4.22 (CH_2_, dd, J = 11.7 Hz, 5.5 Hz; dd, J = 11.7 Hz, 4.0 Hz, 5’_a,b_-H), 3.98 (CH, dd, J = 5.5 Hz, 4.0 Hz, 4’-H), 4.09 (CH, d, J = 10.6 Hz, 14-H), 5.24 (CH, s, 1’-H), 6.98 (CH, dt, J = 8.4 Hz, 2.3 Hz, 11-H), 9.25 (CHO, s, 15-H)

^13^C NMR (CDCl_3_): δ (ppm): 17.5 (CH_3_, C-16), 21.5 (CH_3_, C-19 or C-20), 21.9 (CH_3_, C-19 or C-20), 24.6 (CH_3_, C-17), 26.9 (CH_2_, C-7), 27.2 (CH, C-18), 28.5 (CH_2_, C-2), 29.5 (CH_2_, C-10), 36.4 (CH_2_, C-8), 38.4 (CH_2_, C-1), 41.8 (C-q, C-6), 42.9 (CH, C-5), 45.9 (CH_,_ C-13), 49.6 (C-q, C-9), 68.5 (CH_2_, C-5’), 75.0 (CH, C-4’), 84.0 (C-q, C-2’), 87.4 (CH, C-14), 108.3 (CH, C-1’), 136.1 (CH, C-4), 139.9 (C-q, C-3), 142.4 (C-q, C-12), 159.3 (CH, C-11), 196.0 (CHO, C-15), 201.0 (C-q, C-3’)

**Striatin A:**

^1^H NMR (MeOH-d_4_): δ (ppm): 0.98 (CH_3_, d, J = 6.8 Hz, 19-H or 20-H), 0.99 (CH_3_, d, J = 6.8 Hz, 19-H or 20-H), 1.01 (CH_3_, s, 16-H), 1.06 (CH_3_, s, 17-H), 1.44 – 1.74 (CH_2_, m, 7_a,b_-H), 1.51– 1.57 (CH_2_, m, 8_a,b_-H), 1.57 – 1.63 (CH_2_, 1_a,b_-H), 2.11 (CH_3_CO_2_, s), 2.32 (CH_2_, m, 2-H), 2.43 – 2.64 (CH_2_, m, 10_a,b_-H), 2.54 (CH, d, J = 11.8 Hz, 5-H), 2.86 (CH, sept, J = 6.8 Hz, 18-H), 3.08 (CH, m, 13-H), 3.43 (OCH_3_, s), 3.51 – 3.76 (CH_2_, dd, J = 10.6 Hz, 9.5 Hz; dd, J = 10.6 Hz, 4.7 Hz, 5’_a,b_-H), 4.19 (CH, d, J = 9.9 Hz, 14-H), 4.85 (CH, m, 4’-H), 4.93 (CH, s, 1’-H), 5.20 (CH, m, 15-H), 5.98 (CH, m, 11-H)

^13^C NMR (MeOH-d_4_): δ (ppm): 18.1 (CH_3_, C-16), 21.0 (*C*H_3_CO_2_), 22.1 (CH_3_, C-19 or C-20), 22.4 (CH_3_, C-19 or C-20), 25.2 (CH_3_, C-17), 28.4 (CH, C-18), 28.5 (CH_2_, C-7), 29.3 (CH_2_, C-2), 29.6 (CH_2_, C-10), 37.9 (CH_2_, C-8), 39.7 (CH_2_, C-1), 41.5 (C-q, C-6), 45.1 (CH, C-5), 47.2 (CH_,_ C-3), 50.9 (C-q, C-9), 55.6 (OCH_3_), 61.6 (CH_2_, C-5’), 72.9 (CH, C-4’), 81.8 (C-q, C-2’), 91.4 (CH, C-14), 96.6 (C-q, C-3’), 99.9 (CH, C-15), 107.2 (CH, C-1’), 132.3 (CH, C-11), 134.0 (C-q, C-12), 138.9 (C-q, C-4), 140.6 (C-q, C-3), 171.9 (C-q, CH_3_*C*O_2_)

**Striatin B:**

^1^H NMR (MeOH-d_4_): δ (ppm): 0.95 (CH_3_, d, J = 6.9 Hz, 19-H or 20-H), 1.01 (CH_3_, s, 17-H), 1.01 (CH_3_, d, J = 6.9 Hz, 19-H or 20-H), 1.22 (CH_3_, s, 16-H), 1.43 – 1.73 (CH_2_, m, 7_a,b_-H), 1.51 – 1.69 (CH_2_, m, 1_a,b_-H), 1.56 (CH_2_, m, 8-H), 2.11 (COCH_3_, s), 2.22 (CH, s, 5-H), 2.33 (CH_2_, m, 2-H), 3.01 (CH, dm, J = 9.5 Hz, 13-H), 3.39 (CH, m, 18-H), 3.45 (OCH_3_,s), 3.54 – 3.81 (CH_2_, dd, J = 11 Hz, 9 Hz; dd, J = 11 Hz, 4.7 Hz, 5’_a,b_-H), 4.62 (CH, d, J = 7.5 Hz, 10-H), 4.68 (CH, d, J = 9.5 Hz, 14-H), 4.89 (CH, dd, J = 9 Hz, 4.7 Hz, 4’-H), 4.94 (CH, s, 1’-H), 5.22 (CH, s, 15-H), 6.09 (CH, dm, J = 7.5 Hz, 11-H)

^13^C NMR (MeOH-d_4_): δ (ppm): 20.9 (*C*H_3_CO_2_), 21.7 (CH_3_, C-16), 22.1 (CH_3_, C-19 or C-20), 22.4 (CH_3_, C-19 or C-20), 24.4 (CH_3_, C-17), 26.8 (CH, C-18), 29.4 (CH_2_, C-2), 30.2 (CH_2_, C-7), 38.2 (CH_2_, C-8), 40.6 (CH_2_, C-1), 41.4 (C-q, C-6), 48.0 (CH, C-5), 49.9 (CH, C-13), 50.5 (C-q, C-9), 55.9 (OCH_3_), 61.7 (CH_2_, C-5’), 70.2 (CH, C-10), 72.8 (CH, C-4’), 81.4 (C-q, C-2’), 91.5 (CH, C-14), 96.8 (C-q, C-3’), 99.2 (CH, C-15), 107.1 (CH, C-1’), 134.0 (CH, C-11), 135.9 (C-q, C-12), 138.0 (C-q, C-4), 140.9 (C-q, C-3), 171.8 (C-q, CH_3_*C*O_2_)

**Erinacin P**:

^1^H NMR (CDCl_3_): δ (ppm): 0.93 (CH_3_, s, 17-H), 0.94 (CH_3_, d, J = 6.8 Hz, 19-H,20-H), 0.94 (CH_3_, s, 16-H), 1.43 (CH_2_, m, 8-H), 1.44 – 1.53 (CH_2_, m, 7_a,b_-H), 1.45 – 1.59 (CH_2_, m, 1_a,b_-H), 1.82 – 2.56 (CH_2_, m; dd, J = 14 Hz, 8.3 Hz, 10_a,b_-H), 2.02 (COCH_3_, s), 2.12 (CH, m, 5-H), 2.26 (CH_2_, m, 2-H), 2.73 (CH, sept, J = 6.8 Hz, 18-H), 3.30– 4.02 (CH_2_, dd, J = 11.8 Hz, 9 Hz; dd, J = 11.8 Hz, 5 Hz, 5’_a,b_-H), 3.49 (CH, dd, J = 8 Hz, 6.7 Hz, 2’-H), 3.57 (CH, t, J = 8 Hz, 3’-H), 3.75 (CH, ddd, J = 9 Hz, 8 Hz, 5 Hz, 4’-H), 4.39 (CH, d, J = 6.7 Hz, 1’-H), 4.41 (CH, d, J = 5.3 Hz, 14-H), 5.86 (CH, t, J = 8.3 Hz, 11-H), 6.89 (CH, d, J = 5.3 Hz, 13-H), 9.43 (CHO, s, 15-H)

^13^C NMR (CDCl_3_): δ (ppm): 16.9 (CH_3_, C-16), 21.1 (*C*H_3_CO_2_), 21.5 (CH_3_, C-19 or C-20), 21.8 (CH_3_, C-19 or C-20), 24.5 (CH_3_, C-17), 26.9 (CH, C-18), 28.4 (CH_2_, C-2), 29.4 (CH_2_, C-10), 30.9 (CH_2_, C-7), 36.9 (CH_2_, C-8), 38.3 (CH_2_, C-1), 39.9 (CH, C-5), 44.1 (C-q, C-6), 49.2 (C-q, C-9), 64.9 (CH_2_, C-5’), 69.5 (CH, C-4’), 73.3 (CH, C-2’), 75.3 (CH, C-3’), 85.1 (CH, C-14), 105.3 (CH, C-1’), 136.4 (C-q, C-4), 138.0 (C-q, C-12), 140.2 (C-q, C-3), 156.2 (CH, C-13), 170.4 (C-q, CH_3_*C*O_2_), 191.7 (CHO, C-15)
